# Chitin degradation by *Synechococcus* WH7803

**DOI:** 10.1038/s41598-023-47332-0

**Published:** 2023-11-15

**Authors:** Giovanna Capovilla, Kurt G. Castro, Silvio Collani, Sean M. Kearney, David M. Kehoe, Sallie W. Chisholm

**Affiliations:** 1 Department of Civil and Environmental Engineering, Massachusetts Institute of Technology, Cambridge, MA USA; 2 Department of Fysiologisk Botanik, Umeå Plant Science Centre (UPSC), Umeå University, Umeå, Sweden; 3 Department of Biology, Indiana University, Bloomington, IN USA; 4 Department of Biology, Massachusetts Institute of Technology, Cambridge, MA USA

**Keywords:** Microbiology, Molecular biology, Ecology

## Abstract

Chitin is an abundant, carbon-rich polymer in the marine environment. Chitinase activity has been detected in spent media of *Synechococcus* WH7803 cultures—yet it was unclear which specific enzymes were involved. Here we delivered a CRISPR tool into the cells via electroporation to generate loss-of-function mutants of putative candidates and identified ChiA as the enzyme required for the activity detected in the wild type.

## Introduction

The marine cyanobacterium *Synechococcus* is broadly distributed in the marine environment and is the most abundant group of phytoplankton globally in terms of total biomass^1^. As such, it contributes significantly to ocean primary productivity and the ocean carbon cycle. Progress in understanding their physiology, which is of great interest because of their role in ocean ecosystems, could be significantly advanced by the development and improvement of valid molecular tools.

While these bacteria are considered primarily phototrophic and free-living, *Synechococcus* strains possess chitin degradation genes and can switch from their canonical planktonic lifestyle to living attached to particles, including chitin^2^.

Chitin, an insoluble polymer of β1,4-linked N-acetylglucosamine (GlcNAc), is primarily derived from arthropod exoskeletons and serves as an important carbon and nitrogen source for marine microbial consortia^3–5^. To utilize this carbon source, bacteria must degrade chitin into soluble oligosaccharides via the action of enzymes defined as chitinases, which are divided into categories based on their activity^6^. Endochitinases are chitinases that cleave within the polymer strand of chitin, while exochitinases cleave terminal disaccharides from chitin oligosaccharides^6^. Exochitinases are further characterized as chitobiosidase or β-N-acetylglucosaminidase. The former cleaves dimeric units of GlcNAc from the non-reducing terminal of the polymer, and the latter converts the oligomeric products to GlcNAc monomers^7^.

Both extracellular endochitinase and chitobiosidase activity were detected in cell-free supernatants of axenic *Synechococcus* WH7803 cultures^2^, indicating that the cells secrete active chitinases. However, the specific enzymes involved were unknown. Here, we identify the genes required for chitin degradation and their role while broadening the toolbox available for *Synechococcus* genetic manipulation. We demonstrate that electroporation is a reliable strategy for delivering gene editing tools and that CRISPR-Cpf1 has great potential for obtaining targeted mutation.

## Main

**Figure 1:**
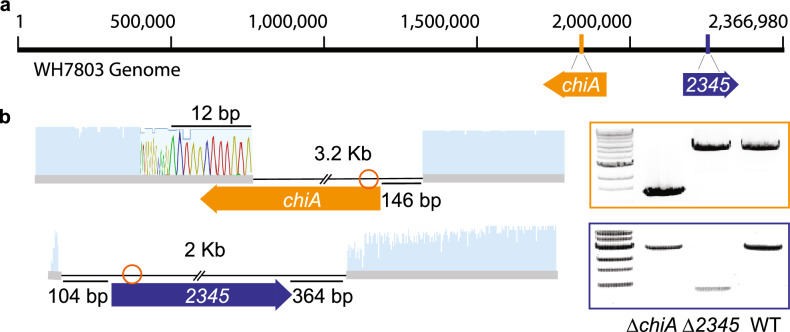
Mutants lacking *chiA* or *2345* obtained with a CRISPR-Cpf1 approach. (**a**) Cartoon representation of the WH7803 genome and the relative positions of the genes of interest. (**b**) Schematic representation of the edited cell lines obtained with the CRISPR-Cpf1 tool. Sanger sequences show the details of each deletion. Orange circles show the location of the PAM sites. PCR products indicate the length of each amplification using primers listed in Table S2, which are designed outside the homologous template regions.

Candidate chitinase genes have been previously identified with bioinformatic tools^2^. A putative chitinase gene in the *Synechococcus* WH7803 genome is *WH7803_2068*, which we refer to as *chiA* henceforth. ChiA contains a Beta-glycosidase of family GH18 listed as a possible chitinase and two N-terminal carbohydrate-binding domain of the CBM2^8^ family (Fig. S1a). We also identified two other proteins of interest—WH7803_2345 and WH7803_2069—which contain two peripheral CBM2 domains and one central CBM2 domain, respectively, with close homology to those in ChiA (Fig. S1a). Their relative position in the *Synechococcus* WH7803 genome is shown in Fig. 1a and Fig. S2a. To determine whether the genes of interest respond to adding chitin to the media, we used qPCR to measure their expression in *Synechococcus* WH7803 cultures grown with and without chitosan or colloidal chitin. The three genes were expressed under all conditions, and their expression increased after adding chitin to the samples, but these increases were not statistically significant (Fig. S1b,c). Consistent with this observation, ChiA was abundant in a previous proteomic analysis even without chitin addition to the growth medium^8^. Similarly, *chiA* is also constitutively expressed in *Prochlorococcus* MIT9303^2^ and other organisms such as diatoms^9^.

To investigate the contribution of each of the three genes to chitin degradation, we designed an approach to obtain and test loss-of-function mutant lines. We employed a CRISPR-Cpf1 plasmid successfully used in freshwater cyanobacteria^10^ to make targeted deletions of each gene. This plasmid contains the CRISPR-Cpf1 cassette, a guide RNA to target a double-strand break on the gene of interest and a homologous repair template that cells can use to repair the DNA via homologous recombination. To deliver the engineered CRISPR plasmids (Table S1), we used an electroporation protocol^11^ with modifications (see methods) rather than a conjugation method, simplifying the recovery and purification of transformants. This work provides a new strategy for modifying cyanobacterial genomes when conjugation is unsuccessful or inefficient, as in the closely related species *Prochlorococcus*^11^.

**Figure 2:**
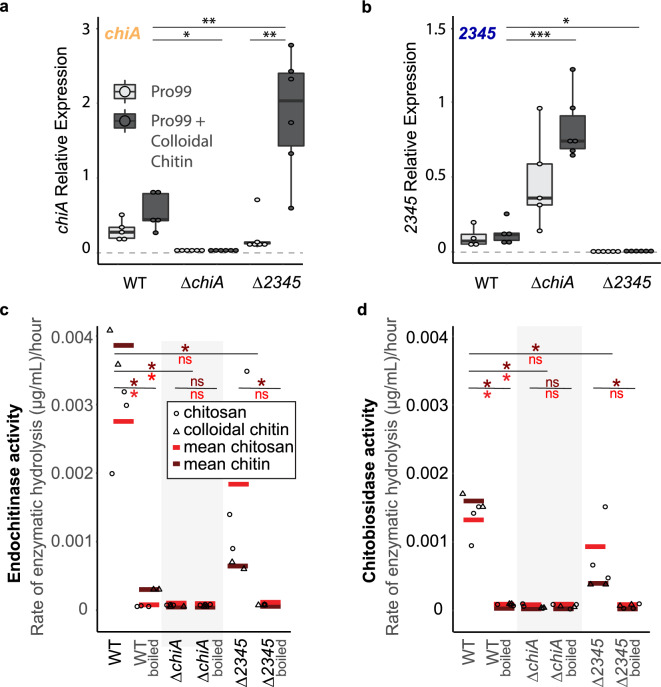
Rate of chitin degradation activity in mutants lacking ChiA or 2345 in comparison to wild-type WH7803. (**a, b**) Expression (measured by qPCR) of *chiA* or *2345* in wild-type and mutant lines in mid-exponential growth in relation to the housekeeping gene, *rnpB,* in natural seawater-based Pro99 medium in presence and absence of colloidal chitin. (**c, d**) Endochitinase and Exochitinase (chitobiosidase) activities measured in wild-type and mutant lines spent media amended with either colloidal chitin or chitosan to a final concentration of 56 μg/ml. Average and statistical significance of activities obtained from chitosan or colloidal chitin addition are shown in red and maroon, respectively. (*P < 0.05, **P < 0.0, *ns* not significant using Welch’s *t* test). Activity is lost after boiling and shown as a negative control for each sample.

The selection of fully edited lines was hampered by polyploidy in WH7803, which carries 3–4 genome copies^12–14^. Several rounds of plating and dilution-to-extinction with selection pressure were required to obtain fully segregating mutants. We ultimately obtained fully edited lines lacking *chiA* or *WH7803_2345*, which we call here *∆chiA* and *∆2345*, respectively (Fig. 1b). Mutants were tested via qPCR to measure the level of expression of the targeted gene (Fig. 2a, b), i.e., to determine if they were true knock-outs. We also obtained a deletion in *WH7803_2069* (Fig. S2), but we were unsuccessful in isolating a fully edited line, so we suspect that 2069 may be beneficial for growth in laboratory conditions. However, the mutant line obtained, *∆2069*, shows a significantly lower expression of *2069* than the wild type (Fig. S2c), thus, we included it in our analysis, considering it a knock-down line.

Once the recovered mutant lines showed growth rates similar to WT (Fig. S3), we tested the endochitinase and chitobiosidase activities reported previously in the WT^2^. We amended our samples with either colloidal chitin or chitosan, a form of chitin that is solubilized through partial deacetylation. Both additions were equally effective in stimulating the chitinase activity in WT cell-free spent media, and the activity disappeared upon boiling the samples (Fig. 2c, d)—consistent with the production of extracellular chitinase enzymes denatured upon heating. *∆chiA* samples displayed neither endochitinase nor chitobiosidase activity, demonstrating that ChiA is required for both chitinase activity (Fig. 2c, d). The* ∆chiA* line showed higher expression of *2345* than in the WT (Fig. 2b). Similarly, expression of *chiA* was higher in *∆2345* than in the WT (Fig. 2a), suggesting that cells lacking one gene compensate by expressing more of the other, which is often the case when proteins work in complexes or have similar functions^15^.

We note that despite the higher expression of the chitinase gene *chiA*, chitinase activity detected in *∆2345* was significantly lower than in the WT (Fig. 2c, d). This result suggests that while not essential for the enzymatic activity, 2345 helps the ChiA enzyme perform the activity and that in its absence, the activity carries on less efficiently. Similarly, chitinase activity in the knock-down line *∆2069* was reduced compared to the WT (Fig. S4). However, in this line, also expression of the chitinase enzyme *chiA* was reduced (Fig. S2c). Therefore, the reduced chitinase activity in *∆2069* is due to a lower expression of c*hiA*, which also results in a higher expression of *2345*, like in *∆chiA*. It is possible that 2069 is involved in regulating *chiA* expression. However, Due to *chiA* proximity to *2069* in the genome (Fig. S2a), the perturbation in *chiA* expression may be due to a disruption in a regulatory region of *chiA* that occurred while obtaining the edited line with the CRISPR-Cpf1 system. To test whether 2345 contributes to the chitinase activity by physically binding to ChiA forming complexes, we generated pETM-11-derived vectors to express these genes in *Escherichia coli* in order to perform an in vitro analysis of the proteins. However, the expression of these genes is lethal to *E. coli*, so testing this hypothesis in vitro was not possible, as no viable colonies were obtained.

Putative chitinases containing chitin-binding domains but lacking glycosyl hydrolase domains have also been described in *Vibrio* and *Serratia* genera as possible adhesins or chitinase regulatory proteins^16,17^. Similarly to our results in *Synechococcus,* their production was induced by presence of chitin^16,18^, and no chitin degradation activity is attributed directly to them^19^. In *Vibrio*, deletion of CBP, a chitin-binding protein, results in a mutant expressing chitinolytic genes constitutively^20^. Likewise, the expression of *chiA* in *Synechococcus*, constitutively expressed in the WT, was found overexpressed in the *∆2345* mutant line (Fig. 2a), suggesting that 2345 also regulates the expression of *chiA*.

Finally, because CBPs have been shown to facilitate chitin colonization in *V. cholerae*^21–23^, we wondered whether *chiA* and *2345* had a similar role in *Synechococcus.* We tested this indirectly by estimating cell adhesion to added colloidal chitin—measuring both bulk fluorescence and cell number in suspension—in the WT and the loss of function mutants (Fig. S5). We used *Prochlorococcus* MED4 as a control, as it does not attach to chitin^2^_._ In all samples, the growth rate calculated based on the bulk fluorescence was not affected by the addition of colloidal chitin (Fig. S5a-d). But all *Synechococcus* lines (WT and mutants) amended with colloidal chitin showed a significant decrease in cell count in suspension by day 4 (Fig. S5 e–h). Cell loss in this planktonic state is due to cells attaching to the chitin polymer. Cells attached contribute to the fluorescence measured but cannot be detected by flow cytometry. We note that there was no appreciable difference in attachment between WT, *∆chiA*, and *∆2345* (Fig. S5e–g), indicating that either the products of these genes are not involved in attachment or that chitin binding is multifactorial in *Synechococcus.* These results are consistent with previous findings showing that *Synechococcus* WH7803 can adhere to other surfaces^2^.

In summary, we show that the CRISPR-Cpf1 system can be delivered via electroporation in *Synechococcus marinus* to generate loss-of-function mutants. We identified *chiA* as the gene required for chitin degradation and 2345 as a protein indirectly involved in regulating its activity. A major bottleneck in better understanding these minimal phototrophs’ physiology is the inability to easily manipulate the cells genetically. This work takes a significant step forward in obtaining a reliable toolbox for *Synechococcus* and, potentially, *Prochlorococcus*.

## Methods

### Culture conditions and growth curves

*Synechococcus* cells were grown under constant light flux at 12 μmol quanta m^−2^ s^−1^ and 24 °C in natural seawater-based Pro99 medium containing 0.2-μm-filtered Sargasso Sea water, amended with Pro99 nutrients (N, P, and trace metals) prepared as previously described^24^. Where indicated, the samples were amended with high molecular weight chitosan or colloidal chitin (Millipore Sigma) to a final concentration of 56 μg/ml.

Growth was monitored using bulk culture fluorescence measured with a 10AU fluorometer (Turner Designs). Cell concentration was measured using a Guava easyCyte 12HT flow cytometer (EMD Millipore, Billerica, MA). Cells were excited with a blue 488 nm laser for measuring chlorophyll fluorescence (692/40 nm).

### Quantitative PCR analysis

*Synechococcus* cells grown at 12 μmol photons m^−2^ s^−1^ were collected by centrifugation. RNA samples were extracted with a standard acidic Phenol:Chloroform protocol and measured with Nanodrop (Thermo Scientific). RevertAid First Strand cDNA Synthesis Kit (Thermo Scientific) with random primers was used to obtain cDNA. Quantitative PCR reactions were performed in a CFX96 thermocycler (Bio-Rad) using the primers listed in Table S2. The expression of *rnpB* gene diluted 1:100 was used to normalize the results.

### Chitinase assay

*Synechococcus* WH7803 wild type and mutant cultures were grown in constant light at 12 μmol quanta m^−2^ s^−1^ in Pro99 media amended with high molecular weight chitosan (#419419 Sigma-Aldrich® Saint Louis, MO, USA) or colloidal chitin (from chitin powder #J61206 Thermo Scientific Alfa Aesar) to a final concentration of 56 μg/ml in triplicates or duplicates, respectively. Cell concentration was measured using a Guava easyCyte 12HT flow cytometer (EMD Millipore, Billerica, MA, USA). Cells were excited with a blue 488 nm laser for measuring chlorophyll fluorescence (692/40 nm). A volume containing 2E + 09 total cell number was calculated and then centrifuged to remove cells from the spent media. The supernatant was filtered through a 0.2 μm filter and concentrated using 30 kDa Amicon® Ultra-15 Centrifugal Filter Units (Millipore, Darmstadt, Germany) to a volume of 1.5 ml. Half the sample volume was boiled at 90 °C for 30 min to serve as control. Each sample was then divided into 3 aliquots. Each aliquot was tested with one of the 3 substrates contained in the Chitinase kit (#CS1030 Sigma-Aldrich® Saint Louis, MO, USA): 4-Methylumbelliferyl N,N′-diacetyl-β-D-chitobioside (substrate suitable for exochitinase activity detection or chitobiosidase activity), 4-Methylumbelliferyl N-acetyl-β-d-glucosaminide (substrate suitable for exochitinase activity detection of β-N-acetylglucosaminidase activity) and 4-Methylumbelliferyl β-D-N,N′,N′′-triacetylchitotriose (substrate suitable for endochitinase activity detection). The aliquots were mixed with these substrates and kept in darkness. The fluorescence of the 4-methyl-umbelliferone released by the chitinase activity in the sample was measured every 2 h on a plate reader set at excitation 360 nm and emission at 450 nm.

### Electroporation and CRISPR plasmid construction

We constructed our vectors using the plasmid pSL2680 and designed the sgRNAs as described^10^. A homologous repair template was synthesized as left and right fragments with 700–750 bp of homology to each gene’s upstream and downstream sequences using the primers listed in Table S2.

Cells in late-exponential phase (~ 10^8^ cell/ml) were pelleted and washed twice in ice-cold osmoprotectant: 0.4 M mannitol (#63560 Merck Life Science UK Limited, Gillingham, Dorset, UK), 1 mM HEPES pH 7.5(#H8651 Merck Life Science UK Limited, Gillingham, Dorset, UK), to remove all traces of seawater.

Cells were then concentrated in 80 µL (~ 10^10^ cells/ml) to which the plasmid of interest was added. Samples were electroporated (2.5 kv, 500 ohms and 25 µF) and resuspended in seawater media. After incubating for 24 h at 10 µE m^−2^ s^−1^, cells were collected by centrifugation and pour-plated in sterile seawater based 0.3% low melting point agarose solution (#16520 Invitrogen™ Carlsbad, CA, USA) heated at 30 °C with the addition of 50 µM kanamycin sulfate (#60615 Merck Life Science UK Limited, Gillingham, Dorset, UK), 10 mM sodium bicarbonate (#S6014 Merck Life Science UK Limited, Gillingham, Dorset, UK), and 1 mM sodium sulfite (#S4672 Merck Life Science UK Limited, Gillingham, Dorset, UK). Plates were transferred to ambient light conditions (12–15 µE m^−2^ s^−1^). Colonies were PCR screened for presence of appropriate deletions. Two rounds of plating or dilution to extinction were performed to obtain fully edited lines.

### In vitro expression vectors

*chiA*, *2345* and *2069* full-length coding sequences were cloned into a pUC19-derived propagation vector for Golden Gate assembly. In order to obtain T7/lac inducible vectors for protein induction, Green-Gate assembly on pETM-11 derived vectors was attempted in both BL21(DE3) or DH5alpha *E. coli* strains. However, we obtained no colonies in the several rounds of assembly attempted.

### Supplementary Information

Supplementary Information 1.

Supplementary Information 2.

Supplementary Information 3.

## Data Availability

All data generated or analysed during this study are included in this published article and its supplementary information files.
