# Supplementary material for: Chitin degradation by Synechococcus WH7803
Source: Sci Rep. 2023 Nov 15;13:19944. doi: 10.1038/s41598-023-47332-0 (PMC10651935; doi:10.1038/s41598-023-47332-0)
Supplement: Supplementary file 1 — Supplementary Information 1. [file 41598_2023_47332_MOESM1_ESM.pdf]

Chitin degradation by *Synechococcus* WH7803

Giovanna Capovilla*[1], Kurt G. Castro[1], Silvio Collani[2], Sean M. Kearney[1], David M. Kehoe[3], Sallie W. Chisholm*[1,4]

[1]Department of Civil & Environmental Engineering, Massachusetts Institute of Technology, Cambridge, MA, USA

[2]Department of Fysiologisk Botanik, Umeå University (UPSC), Umeå, Sweden

[3]Department of Biology, Indiana University, Bloomington, IN, USA

[4]Department of Biology, Massachusetts Institute of Technology, Cambridge, MA, USA

* Corresponding authors
Giovanna Capovilla (gio.capovilla@gmail.com)
Sallie W. Chisholm (chisholm@mit.edu)

Supplementary Figures

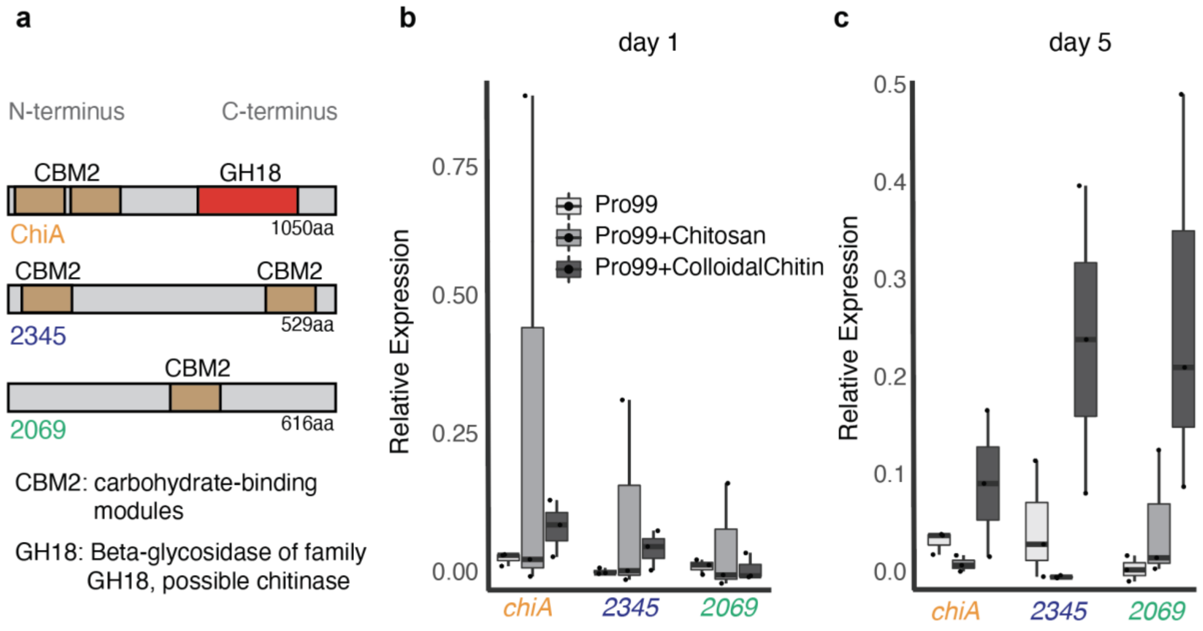

Fig.S1: Identification of the genes of interest.
a, Cartoon representation of domain structure of relevant exoproteins detected in marine *Synechococcus* WH7803. The number of amino acids is reported for each. Carbohydrate-binding domains (CBM2) are reported in brown, and Beta-glycosidase domains (GH18) in red. **b-c**, Expression (measured by qPCR) of *chiA*, *2345* or *2069* in wild-type in mid-exponential growth in relation to the housekeeping gene, *rnpB*, in natural seawater-based Pro99 medium in presence and absence of colloidal chitin or chitosan.

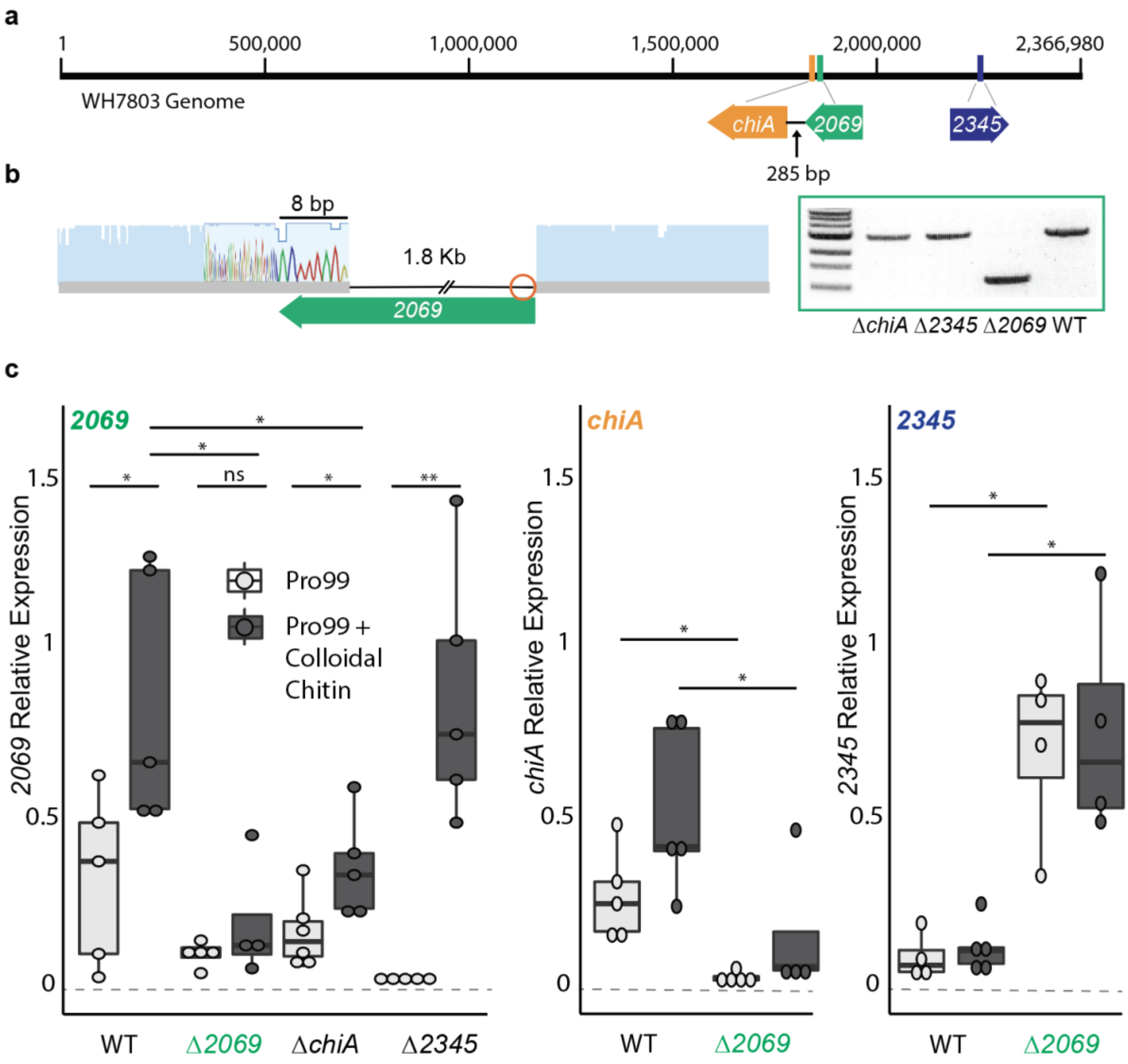

Fig.S2: Mutant lacking *2069*

a, Cartoon representation of the WH7803 genome and the relative positions of all the three genes of interest. **b**, Schematic representation of the edited cell line obtained with the CRISPR-Cpf1 tool. Sanger sequences show the details of each deletion. Orange circles show the location of the PAM sites. PCR products indicate the length of each amplification using primers listed in Table S2. Because of the difference in size, the wild-type band in Δ*2069* is non-detectable with this set of primers. **c**, Expression (measured by qPCR) of *2069*, *chiA*, or *2345* in wild-type and mutant lines in mid-exponential growth in relation to the housekeeping gene, *rnpB*, in natural seawater-based Pro99 medium in presence and absence of colloidal chitin. Expression of *2069* in Δ*2069* was significantly lower than in the WT (*P < 0.05, **P < 0.01, ns= not significant using Welch's t-test).

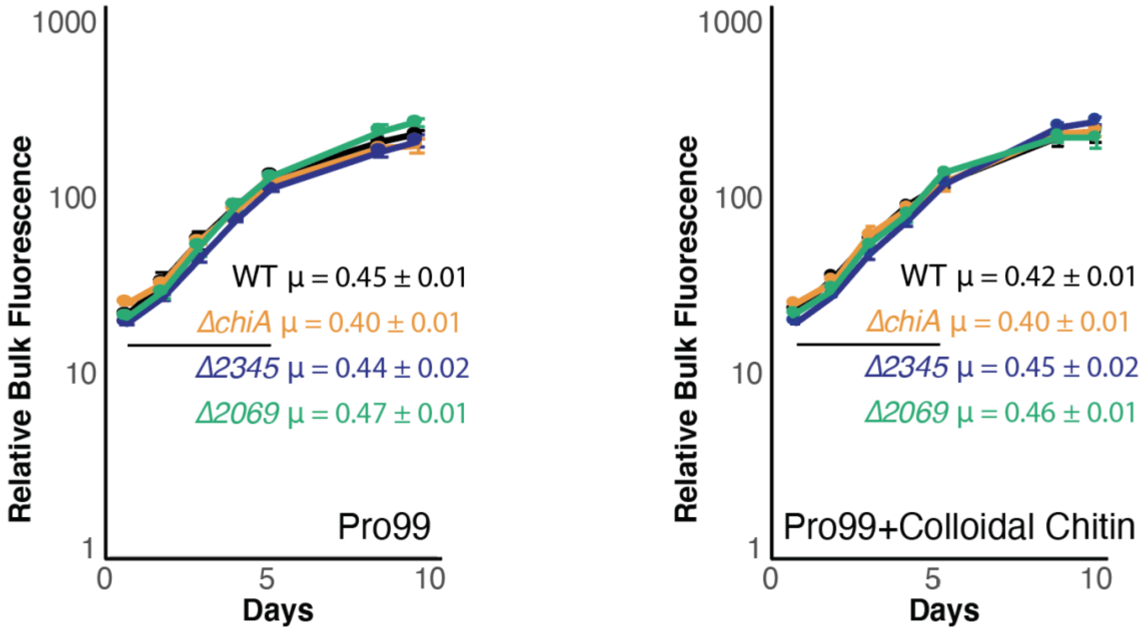

Fig.S3: Growth rates in *Synechococcus* WT and mutant lines.
Growth of *Synecococcus* WH7803 WT and recovered mutant lines in continuous light (at 12 μmol photons m^{-2} s^{-1}) monitored by relative bulk culture chlorophyll fluorescence. Different colors show the average growth for each line in Pro99 media or Pro99 media amended with colloidal chitin. Growth rates and associated standard deviation (μ, in units day^{-1}) was calculated in exponential phase (marked with a black line) and is shown for each curve.

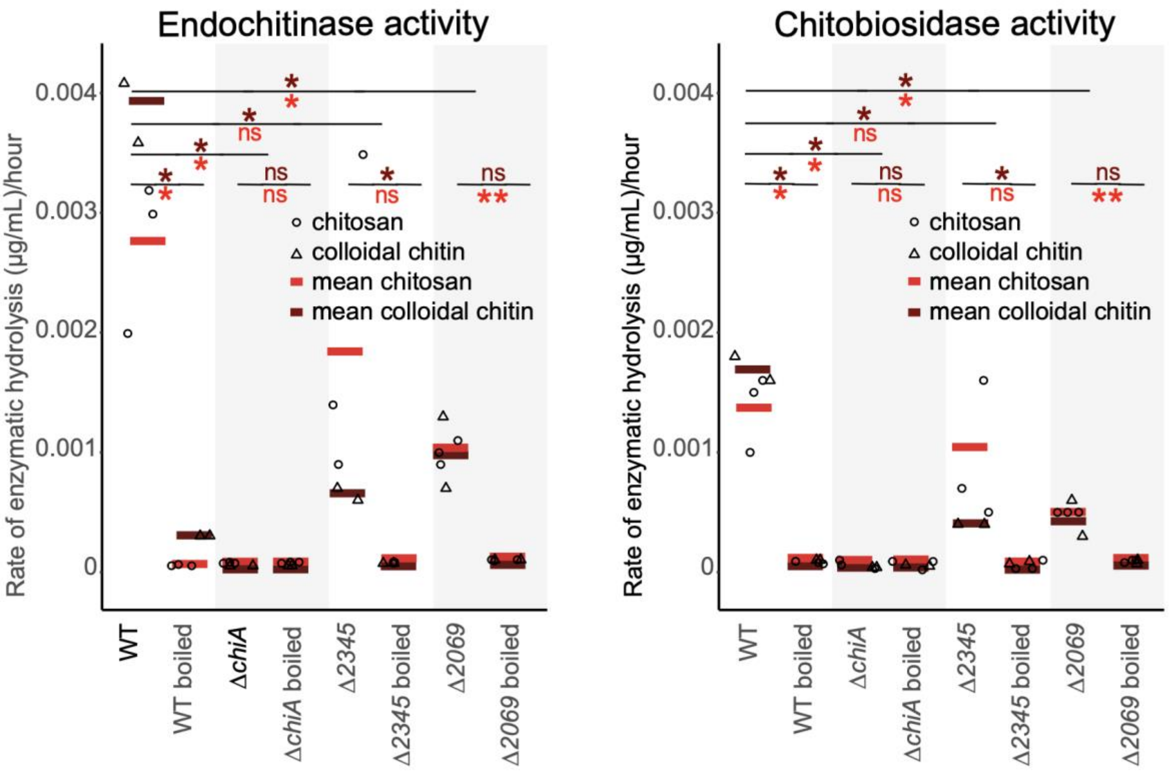

Fig.S4: Chitin degradation activity

Endochitinase and Exochitinase (chitobiosidase) activities measured in wild-type and mutant lines spent media amended with either colloidal chitin or chitosan to a final concentration of 56 µg/ml. Average and statistical significance of activities obtained from chitosan or colloidal chitin addition are shown in red and maroon, respectively (*P < 0.05, **P < 0.01, ns= not significant using Welch's t-test). Activity is lost after boiling and shown as a negative control for each sample. Data relative to WT, Δ*chiA*, and Δ*2345* is also reported in Figure 2a-b.

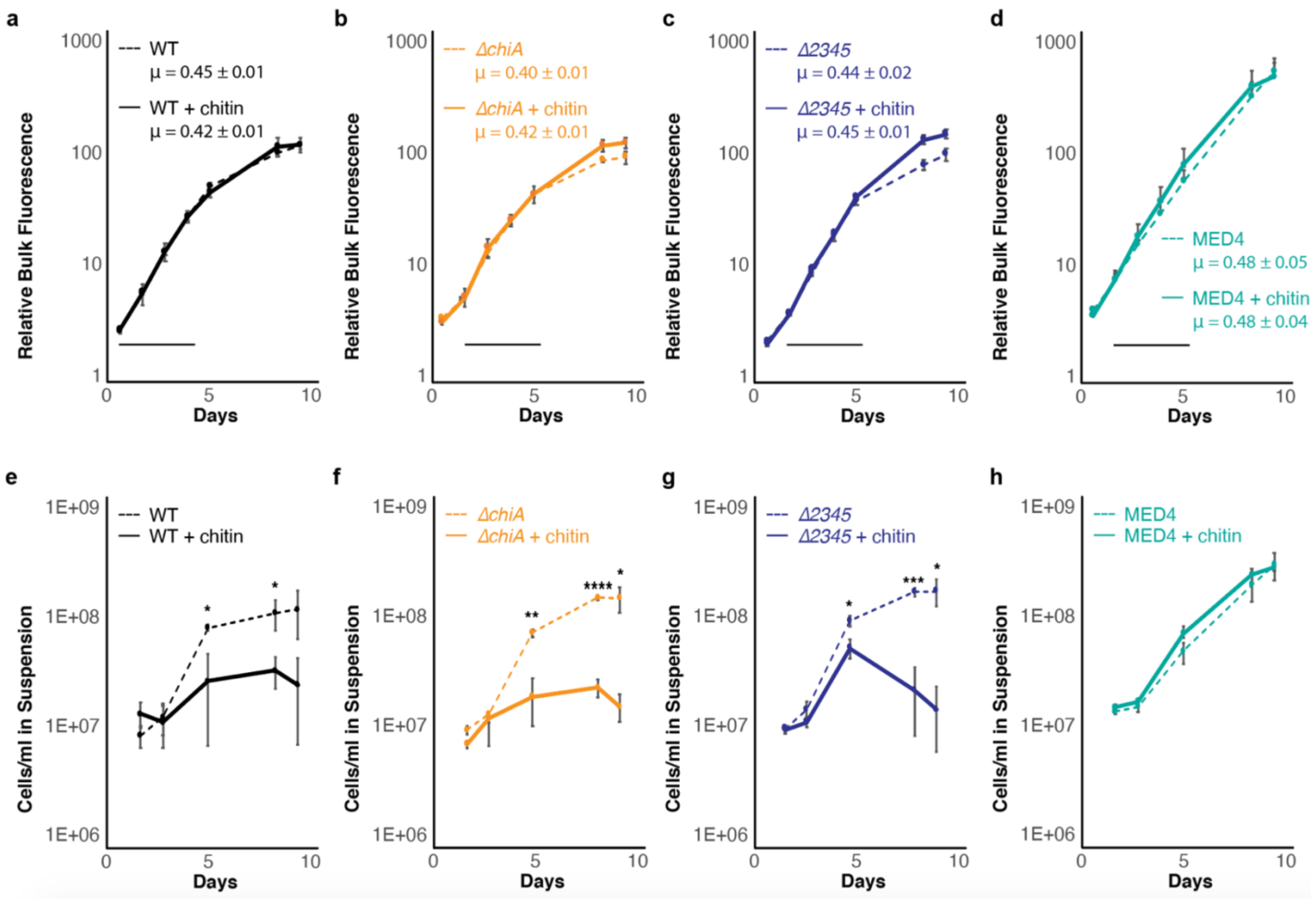

Fig.S5: Adhesion of cells to colloidal chitin particles

Cultures were grown in Pro99 media with (solid line) and without (dashed line) added colloidal chitin. Growth was monitored by bulk chlorophyll fluorescence **a-d**, and cells in suspension were measured using flow cytometry **e-h**. Error bars show standard deviation between three biological replicates. The average growth rate and associated standard deviation (μ, in units day^{-1}) was calculated in exponential phase (marked with a black line) and is shown for each curve. While growth rates between treatments did not differ, *Synechococcus* cells in suspension were less abundant in presence of chitin, as cells attaching to the polymer avoid detection via flow-cytometer. MED4, a high-light adapted *Prochlorococcus* ecotype, does not stick to chitin[2], and consistently no difference was found in cells in suspension between the two treatments. (*P < 0.05, **P < 0.01, ***P < 0.001, ****P < 0.0001 using Welch's t-test)

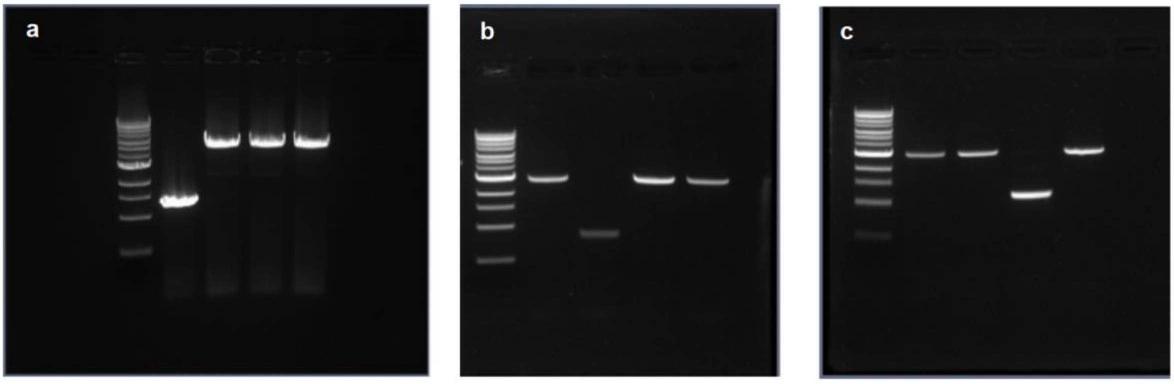

Fig.S6: Uncropped gel blots

Original gel images of PCR products indicating the length of each amplification using primers listed in Table S2. **a)** PCR amplification using primers Δ*chiA*_F and Δ*chiA*_R on samples Δ*chiA*, Δ*2345, WT* and Δ*2069* (also shown in figure 1b) **b)** PCR amplification using primers Δ*2345*_F and Δ*2345*_R on samples Δ*chiA*, Δ*2345, WT* and Δ*2069* (also shown in figure 1b) **c)** PCR amplification using primers Δ*2069*_F and Δ*2069*_R on samples Δ*chiA*, Δ*2345, Δ2069 and WT* (also shown in figure S2b)

Table S1: List of plasmids used

pSL2680_*chiA* KanR CRISPR/Cpf1 plasmid containing gRNA and homologous repair template designed to target *chiA*
pSL2680_*2345* KanR CRISPR/Cpf1 plasmid containing gRNA and homologous repair template designed to target *2345*
pSL2680_*2069* KanR CRISPR/Cpf1 plasmid containing gRNA and homologous repair template designed to target *2069*

Table S2: List of primers used

**PRIMERS LIST**	
**NAME**	**SEQUENCE (5'-3')**
**Primers for cloning pSL2680_*chiA*, pSL2680_*2345* and pSL2680_*2069***	
**HRTL_F_*chiA***	**CATTTTTTTGTCTAGCTTTAATGCGGTAGTTGGTACGACCAGAGCGCACCAATGTA**
**HRTL_R_*chiA***	**ATTCAATGCCGCAAGGAATGAGTCATCTCTTTGCCTGACCAAGCC**
**HRTR_F_*chiA***	**TAGGGGGCTTGGTCAGGCAAAGAGATGACTCATTCCTTGCGGCAT**
**HRTR_R_*chiA***	**CGCTGCCCGGATTACAGATCCTCTAGAGTCGACGGGTTCACTGACTCGGAGCAA**
**gRNA_F_*chiA***	**AGATCACTGGCCTGCAGAAGCAGG**
**gRNA_R_*chiA***	**AGACCCTGCTTCTGCAGGCCAGTG**

**HRTL_F_*2345***	**CATTTTTTTGTCTAGCTTTAATGCGGTAGTTGGTACGTAGCGAGTGATCTGGAGCG**
**HRTL_R_*2345***	**ATCAACACGCCTGAGATCGCTAAGTATGGGGTCTTTCGGCTTACC**
**HRTR_F_*2345***	**GAATGGGTAAGCCGAAAGACCCCATACTTAGCGATCTCAGGCGTG**
**HRTR_R_*2345***	**CGCTGCCCGGATTACAGATCCTCTAGAGTCGACGGCAAAGGCCATGAACAACGT**
**gRNA_F_*2345***	**AGATCACCGCCGAAATCGCCGTAC**
**gRNA_R_*2345***	**AGACGTACGGCGATTTCGGCGGTG**
**HRTL_F_*2069***	**CATTTTTTTGTCTAGCTTTAATGCGGTAGTTGGTACTACACATCAACCGCGAAAAC**
**HRTL_R_*2069***	**ACGGGGCCTTGAGCAACATGAATTCCTATTTGAGGGCGTTCATCG**
**HRTR_F_*2069***	**CGAGGCGATGAACGCCCTCAAATAGGAATTCATGTTGCTCAAGGCC**
**HRTR_R_*2069***	**CGCTGCCCGGATTACAGATCCTCTAGAGTCGACGCGCTTGTGGCTGATCTCAAG**
**gRNA_F_*2069***	**AGATCCGACTTTGATGAGAGCCAT**
**gRNA_R_*2069***	**AGACATGGCTCTCATCAAAGTCGG**

Primers for screening mutant lines

**Δ*chiA*_F**	**CGACAAGGGCTGGTTGATCT**
**Δ*chiA*_R**	**AGAGCTCACTGACGTTTCGG**
**Δ*2345*_F**	**CTGCTGATTCATGGCTTGGC**
**Δ*2345*_R**	**TGATGCGATGGAGATGAGGC**
**Δ*2069*_F**	**TGACTCATTCCTTGCGGCAT**
**Δ*206*9_R**	**TGACGATTCCTTCCGCACTC**

Primers for qPCR

***chiA*_F**	**CCGACAGTTTGGACTTTGGT**
***chiA*_R**	**GTCATCTTCTGGGCGTCATT**
***2345*_F**	**ACCAACACCCTGAAGATGGA**
***2345*_R**	**GTGAGGTGGTTGATGTCGTG**
***2069*_F**	**GTGATCAGTTCGGAGCCAAT**

***2069*_R**	**CCTCCTGGATCTGATCGAAA**
***rnpB*_F**	**CTCTTACCGCACCTTTGCAC**
***rnpB*_R**	**GGGTGACCGTGAGGAGAGT**
